# Supplementary material for: NLRP11 attenuates Toll-like receptor signalling by targeting TRAF6 for degradation via the ubiquitin ligase RNF19A
Source: Nat Commun. 2017 Dec 7;8:1977. doi: 10.1038/s41467-017-02073-3 (PMC5719394; doi:10.1038/s41467-017-02073-3)
Supplement: Supplementary file 1 — Supplementary Information [file 41467_2017_2073_MOESM1_ESM.pdf]

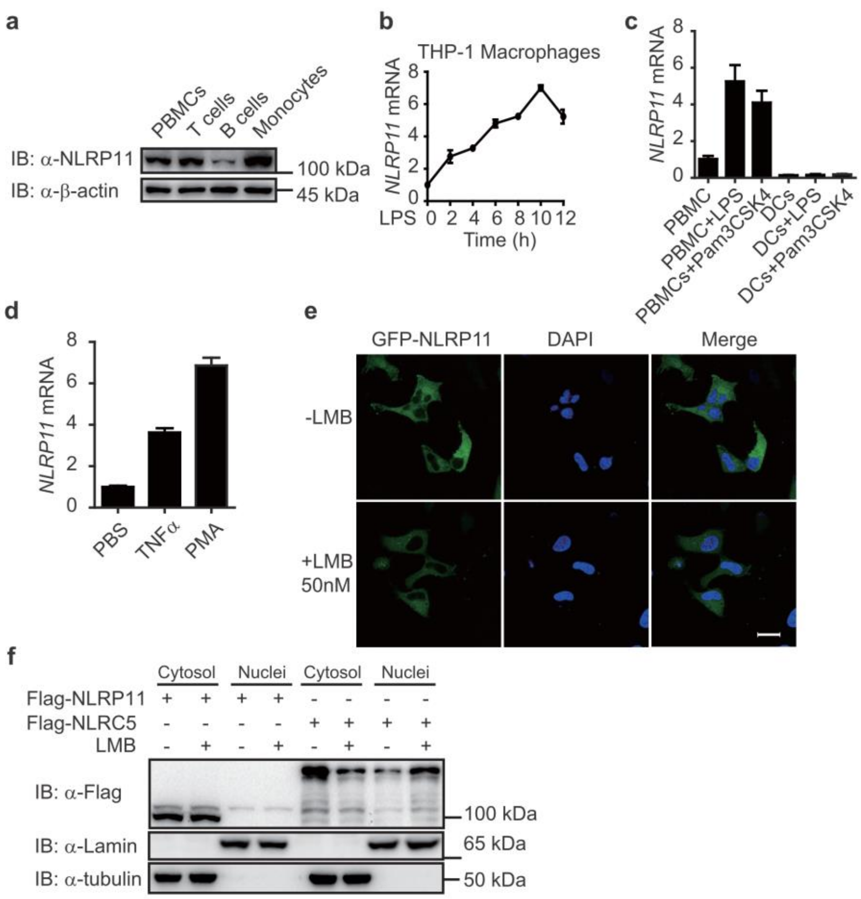

Supplementary Figure 1. Expression and localization of NLRP11. Related to Figure 1. (**a**) Immunoblot analysis of NLRP11 in PBMCs, T cells, B cells, and monocytes. (**b**) THP-1 derived-macrophages were stimulated with LPS (100 ng/ml) for the indicated time points, and then *NLRP11* mRNA was measured by real-time PCR. (**c**) Real-time PCR analysis of NLRP11 expression in PBMCs or DCs in response to LPS (100ng/ml) or Pam3CSK4 (100ng/ml). (**d**) Real-time PCR analysis of NLRP11 in THP-1 cells in response to TNF (10ng/ml) or PMA (200ng/ml) treatment. (**e**) HeLa cells transfected with GFP-NLRP11 were treated with 20 nM leptomycin B (LMB) for 4 h or left untreated. The nuclei were stained by DAPI. Scale bar, 20 μM. (**f**) Cytoplasmic and nuclear extracts of HeLa cells that were transfected with either Flag-NLRP11 or Flag-NLRC5 were analyzed by immunoblotting with antibodies to Flag, tubulin (cytoplasmic fraction), and Lamin (nuclear fraction). Data (**b-d**) are plotted as mean ± SEM of combined from three independent experiments with triplicate. Data (**a, f**) are representatives of three independent experiments with similar results. Representative blots are shown.

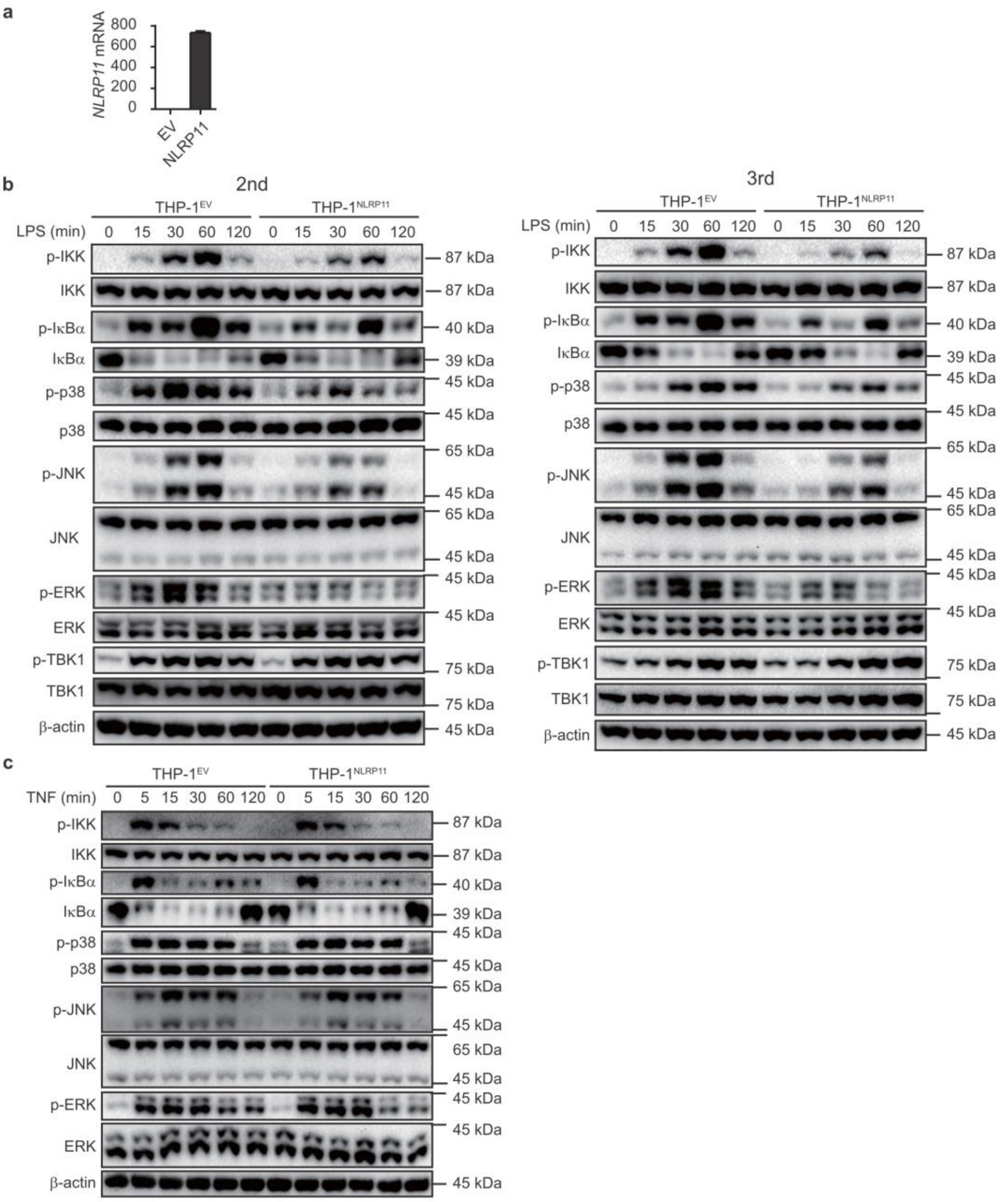

Supplementary Figure 2. NLRP11 inhibits LPS-induced activation of NF-κB and MAPKs. Related to Figure 2. (**a**) Real-time PCR analysis of *NLRP11* expression in THP-1 cells stably expressing empty vector (THP-1^{EV}) or Flag-tagged NLRP11 (THP-1^{NLRP11}). (**b, c**) THP-1^{EV} and THP-1^{NLRP11} cells were stimulated with LPS (100 ng/ml) **(b)** or TNF (10 ng/ml) **(c)** for the indicated periods, then analyzed by immunoblot with indicated antibodies. Data (**a**) are plotted as mean ± SEM of combined three independent experiments with triplicate. Data (**b,c**) are representatives of three independent experiments with similar results.

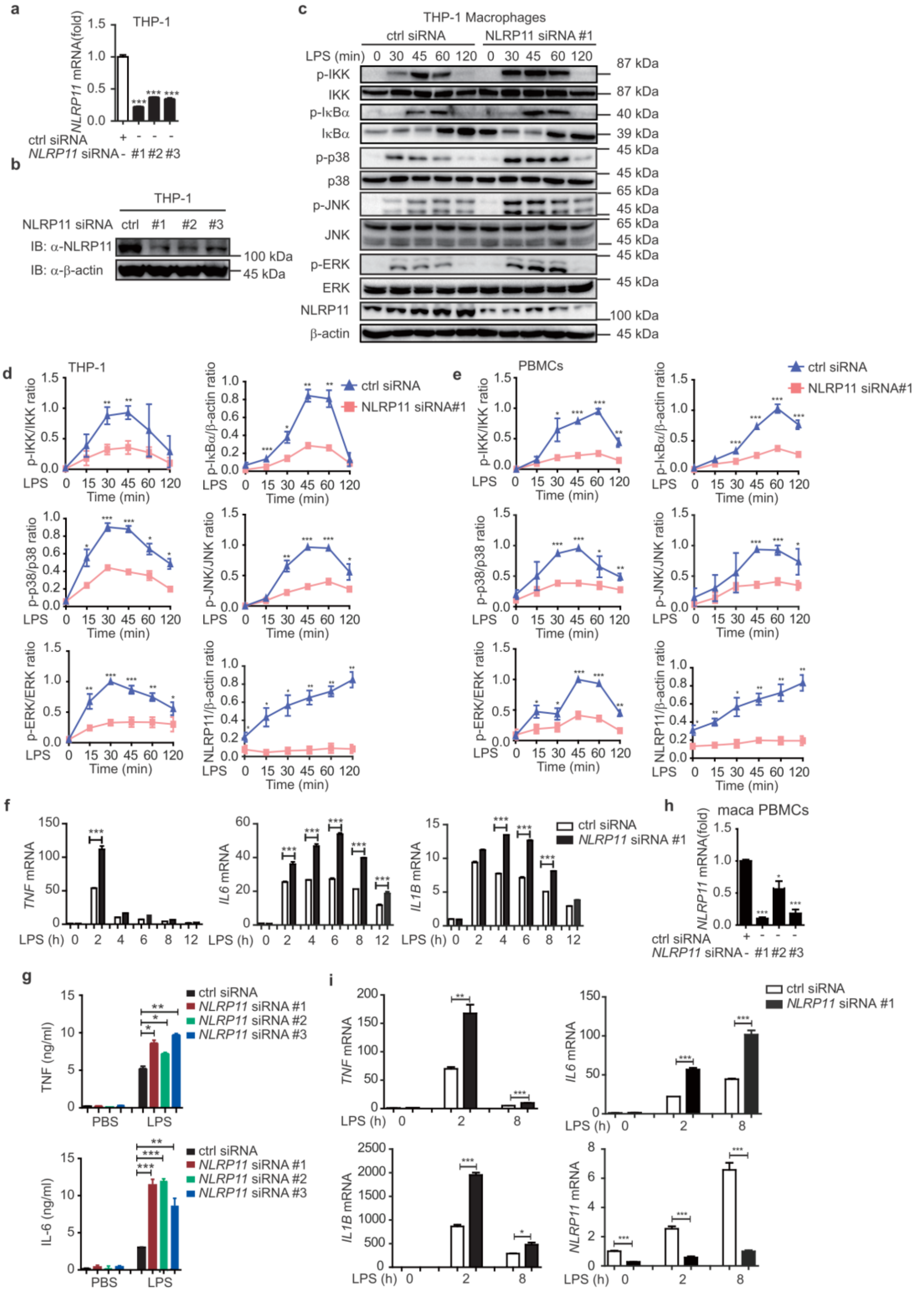

Supplementary Figure 3. NLRP11 knockdown enhances LPS-induced NF-κB and MAPK signalling. Related to Figure 3. (**a**) THP-1 cells were transfected with siRNA targeting different NLRP11 regions or a control siRNA. The knockdown efficiency was analyzed by real-time PCR. (**b**) THP-1 cells were transiently transfected with control

(ctrl) or *NLRP11*-specific siRNA for 48 h. NLRP11 protein was examined by immunoblot. (**c**) Immunoblot analysis of total and phosphorylated IKK, IκBα as well as MAPKs (p38, JNK, ERK) in THP-1 derived-macrophages transfected with indicated siRNA and stimulated with LPS for indicated times. (**d, e**) Quantitative comparison of signalling activation by density scanning of the blots in Figure 3a **(d)** and 3b **(e)**. (**f**) THP-1 derived-macrophages were transfected with control siRNA or *NLRP11*-specific siRNA for 48 h and then stimulated with LPS (100 ng/ml) for indicated periods. Levels of *TNF*, *IL6*, and *IL1B* were determined by real-time PCR analysis, and the values were normalized to *ACTB* mRNA expression. (**g**) THP-1 cells transfected with the indicated siRNA for 36 h were treated with LPS for 24 h then analyzed by ELISAs for secreted TNF and IL-6. (**h**) Real-time PCR analysis of *NLRP11* siRNA knockdown efficiency in Rhesus macaca monkey PBMCs. (**i**) Rhesus macaca monkey PBMCs that were transfected with *NLRP11* siRNA or control siRNA for 48 h were stimulated with LPS (100 ng/ml) for indicated periods, then analyzed by real-time PCR for *TNF*, *IL6*, *IL1B*, and *NLRP11* transcripts. Data (**a**, **f-i**) are plotted as mean ± SEM of combined from three independent experiments triplicate. Data (**b,c**) are representatives of three independent experiments with similar results. Data (**d**) are shown as means ± SEM of combined from three independent experiments. *$P < 0.05$, **$P < 0.01$, and ***$P < 0.001$ versus control using Student's t-test.

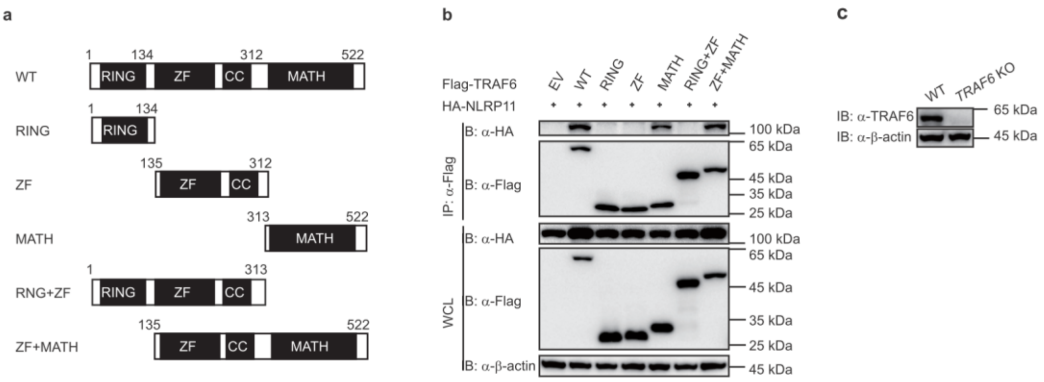

Supplementary Figure 4. NLRP11 interacts with the MATH domain of TRAF6. Related to Figure 6. (**a**) A schematic diagram of TRAF6 and its mutants. (**b**) 293T cells were transfected with the indicated expression vectors. At 24 h after the transfection, cell extracts were analyzed by immunoprecipitation with an anti-Flag antibody followed by immunoblotting with the indicated antibodies. (**c**) Immunoblotting analysis of cell extracts from wild-type (WT) and TRAF6 knockout (KO) 293T cells with the indicated antibodies. Data (**b** and **c**) are representatives of three independent experiments with similar results.

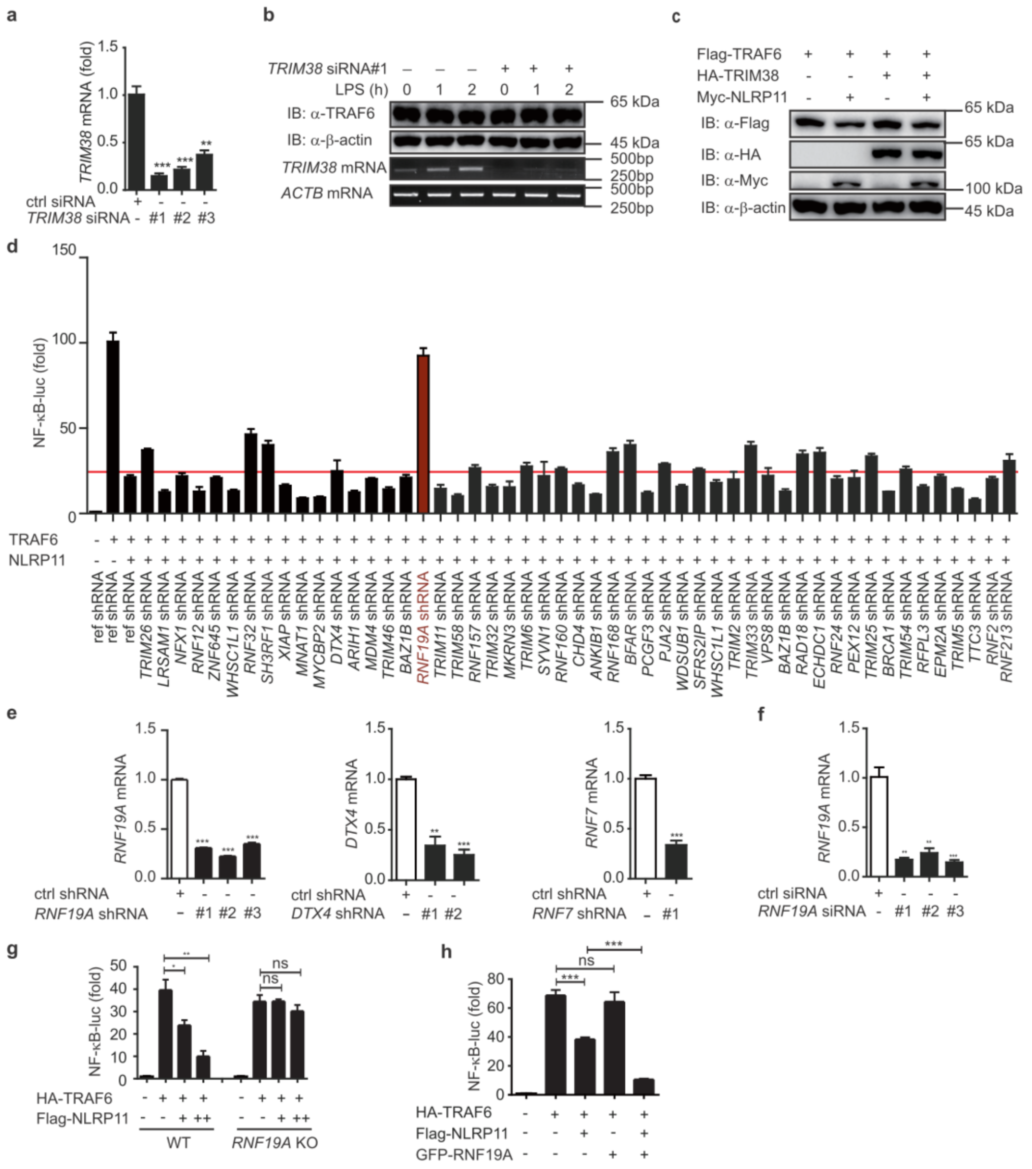

Supplementary Figure 5. NLRP11 recruits RNF19A to degrade TRAF6. Related to Figure 7. (**a**) Real-time PCR analysis of *TRIM38* siRNA knockdown efficiency in THP-1 derived-macrophages. (**b**) Immunoblot analysis of extracts of THP-1 derived-macrophages transfected with *TRIM38* siRNA or control siRNA and simulated with LPS for indicated periods. Bottom, RT-PCR analysis of *TRIM38* mRNA. (**c**) Immunoblot analysis of 293T cells transfected with indicated combinations of expression plasmids. (**d**) 293T cells were first transfected with indicated shRNA vector from a human RING domain-containing E3 ubiquitin ligase shRNA sub-library for 12

h, then the cells were transfected with HA-tagged TRAF6, Flag-tagged NLRP11 and NF-κB reporter. 24 h post the second transfection, the cell lysates were subjected to dual-luciferase assay. (**e**) 293T cells were transfected with the indicated shRNA for 48 h. Knockdown efficiency was analyzed by real-time PCR. (**f**) Real-time PCR analysis of *RNF19A* siRNA knockdown efficiency in PBMCs. (**g**) A luciferase assay of lysates of WT and *RNF19A* KO 293T cells transfected with the indicated plasmids. (**h**) A NF-κB luciferase assay in 293T cells transfected with HA-TRAF6 and Flag-NLRP11 or GFP-RNF19A as indicated. Data (**a**, **d-h**) are plotted as mean ± SEM of combined from three independent experiments with triplicate. Data (**b**,**c**) are representatives of three independent experiments with similar results. *$P < 0.05$, **$P < 0.01$, and ***$P < 0.001$ versus control using Student's t-test.

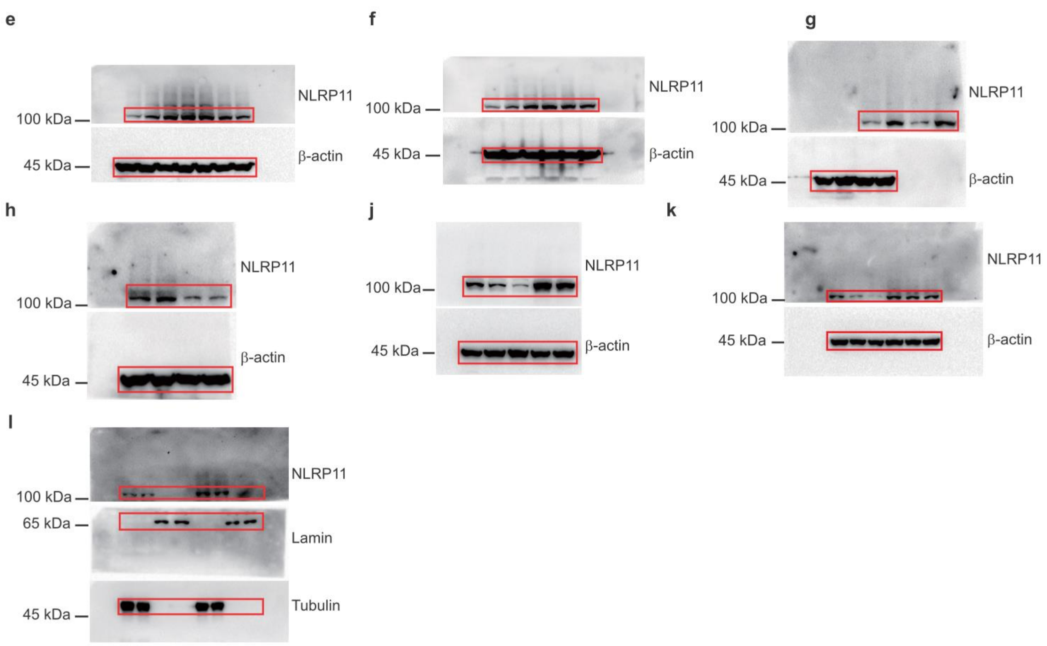

Supplementary Figure 6. Full-length uncropped western blots for figure 1.

Cropped areas are marked by red box.

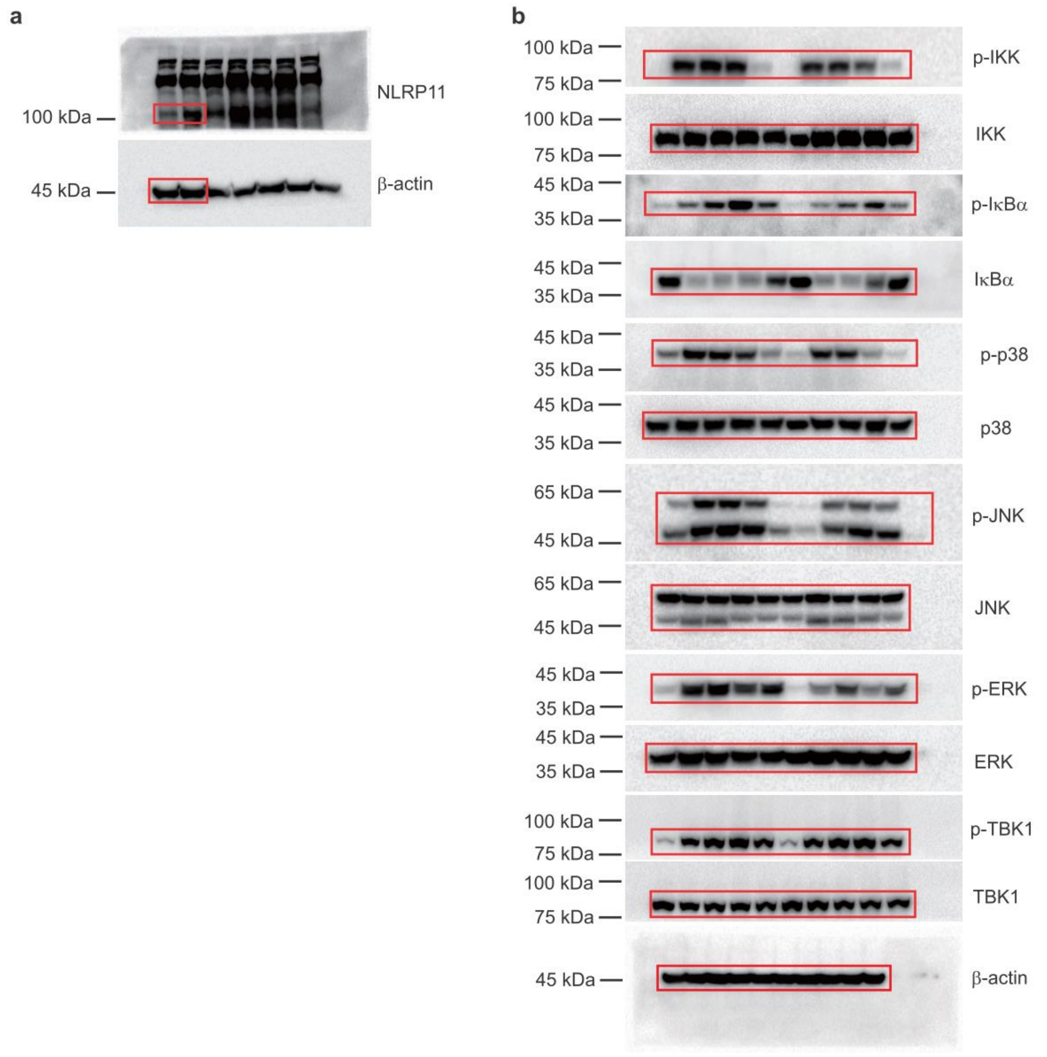

Supplementary Figure 7. Full-length uncropped western blots for figure 2.

Cropped areas are marked by red box.

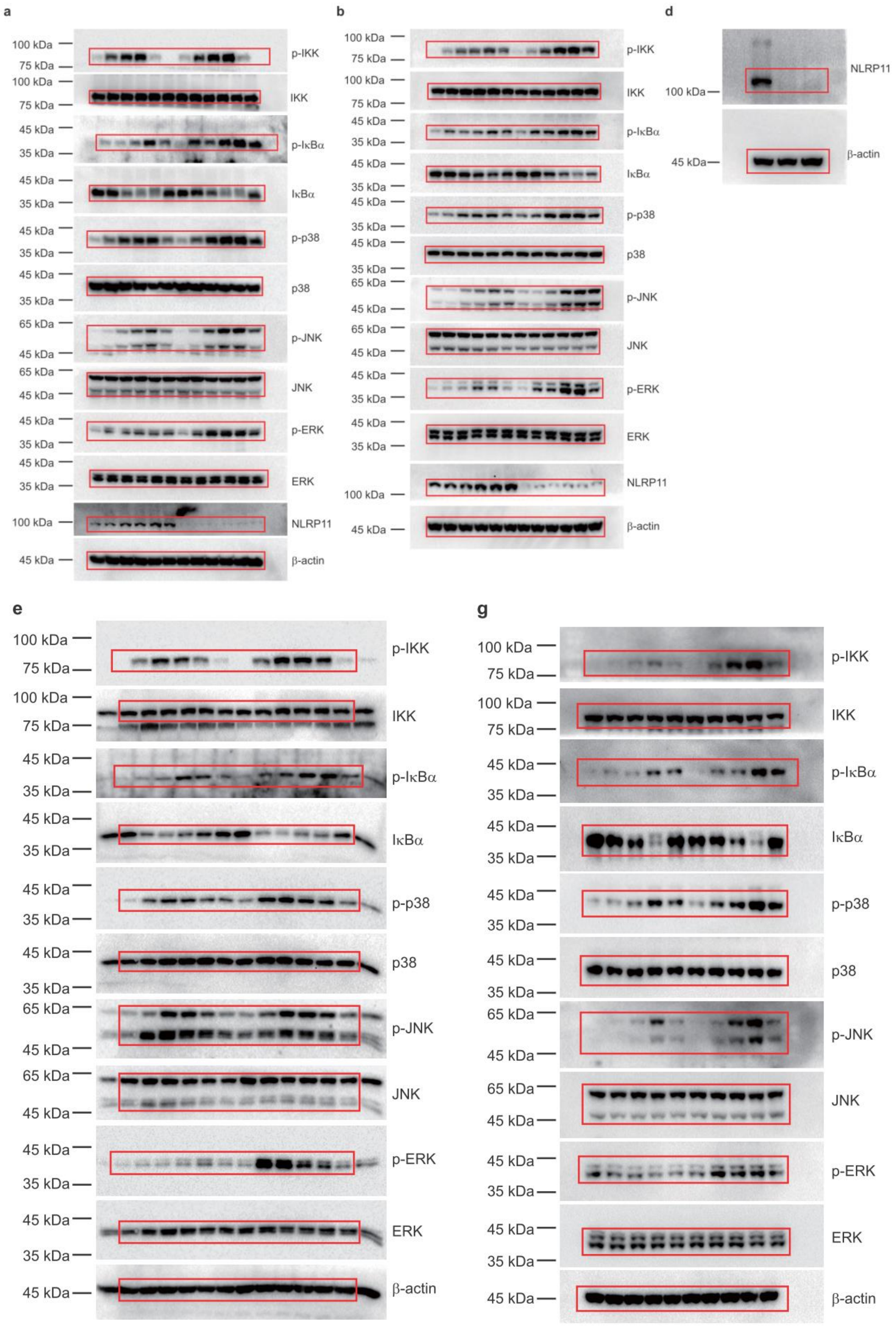

Supplementary Figure 8. Full-length uncropped western blots for figure 3.

Cropped areas are marked by red box.

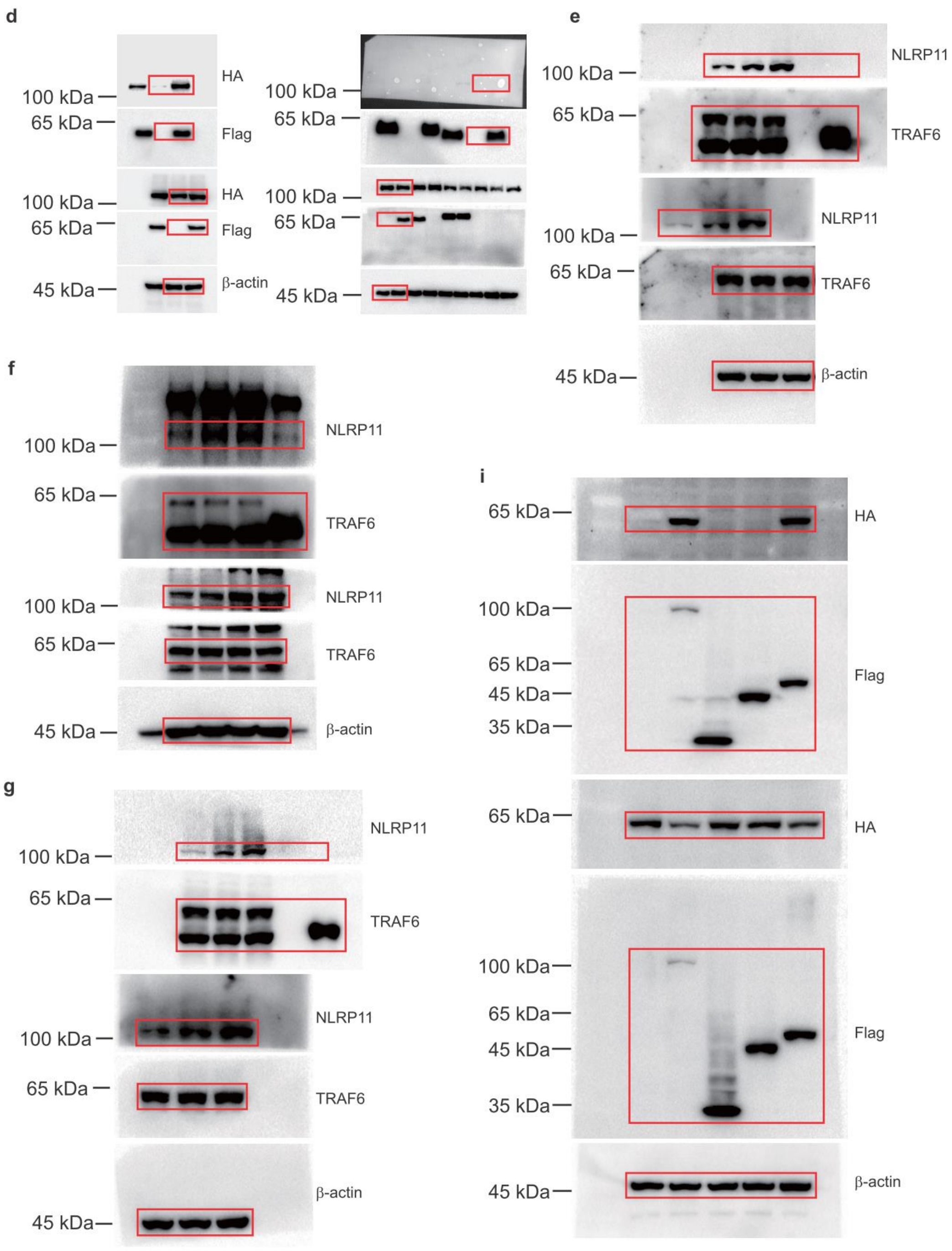

Supplementary Figure 9. Full-length uncropped western blots for figure 4.

Cropped areas are marked by red box.

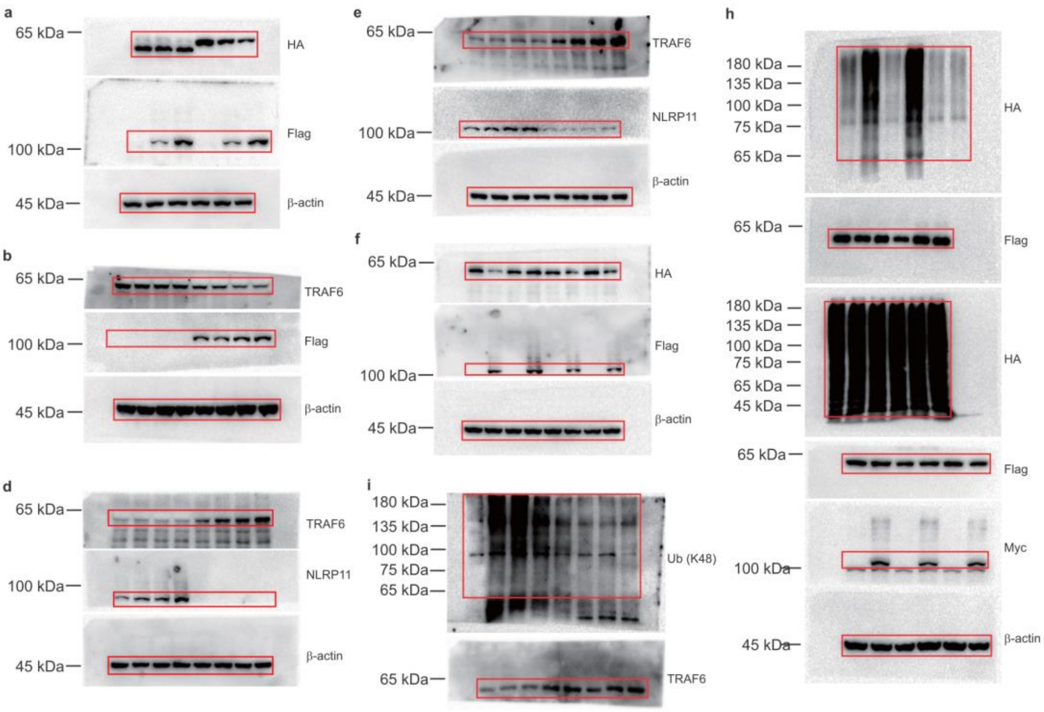

Supplementary Figure 10. Full-length uncropped western blots for figure 5.

Cropped areas are marked by red box.

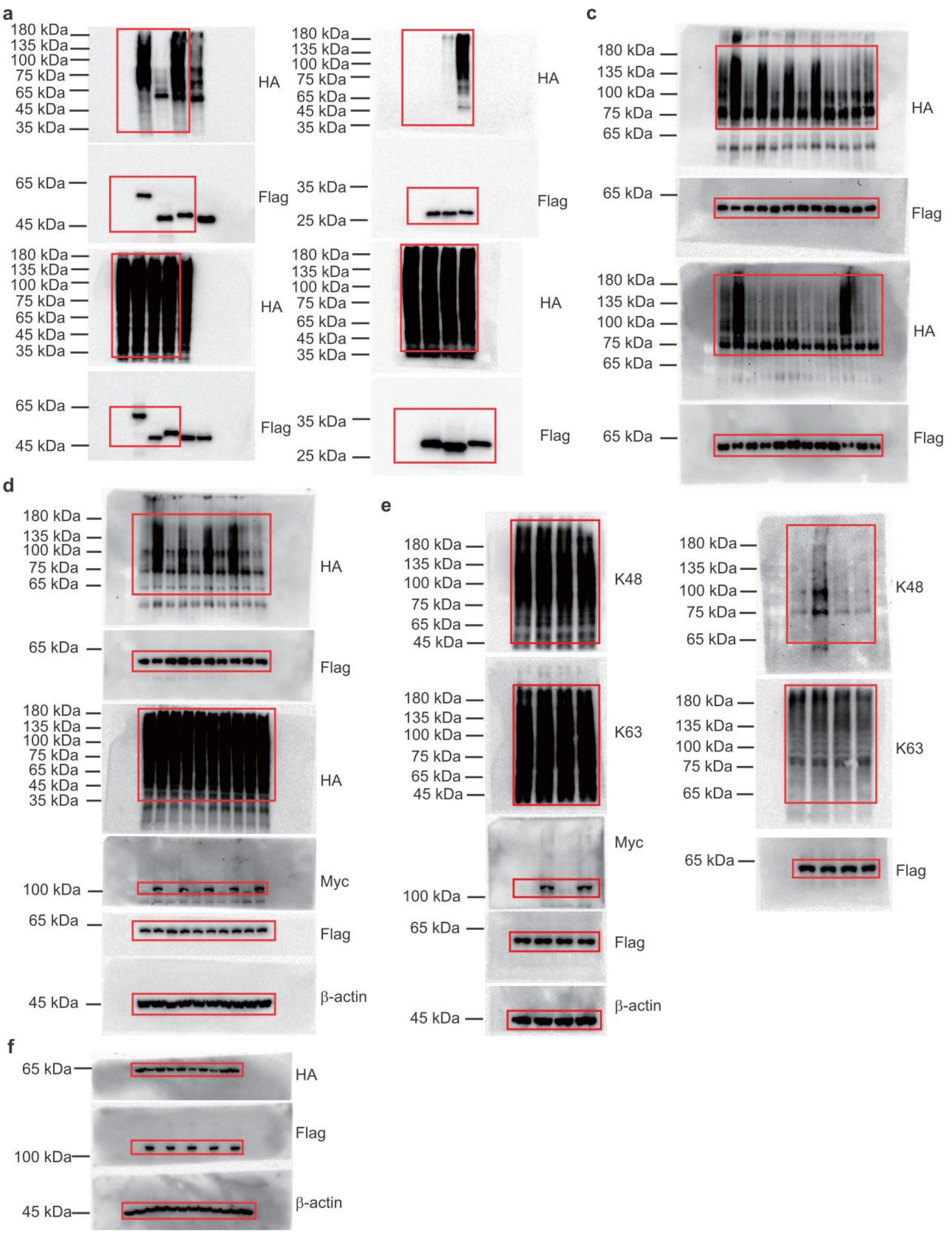

Supplementary Figure 11. Full-length uncropped western blots for figure 6.

Cropped areas are marked by red box.

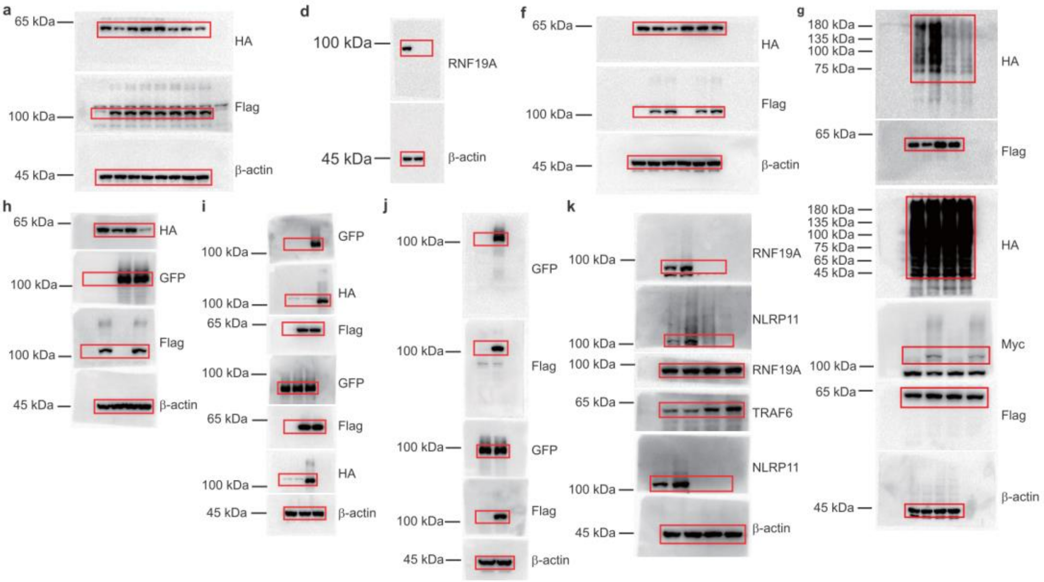

Supplementary Figure 12. Full-length uncropped western blots for figure 7.

Cropped areas are marked by red box.

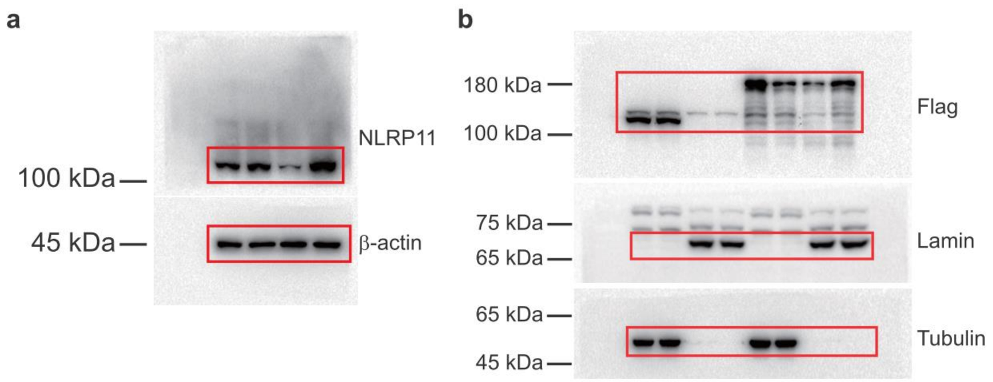

Supplementary Figure 13. Full-length uncropped western blots for supplementary figure 1. Cropped areas are marked by red box.

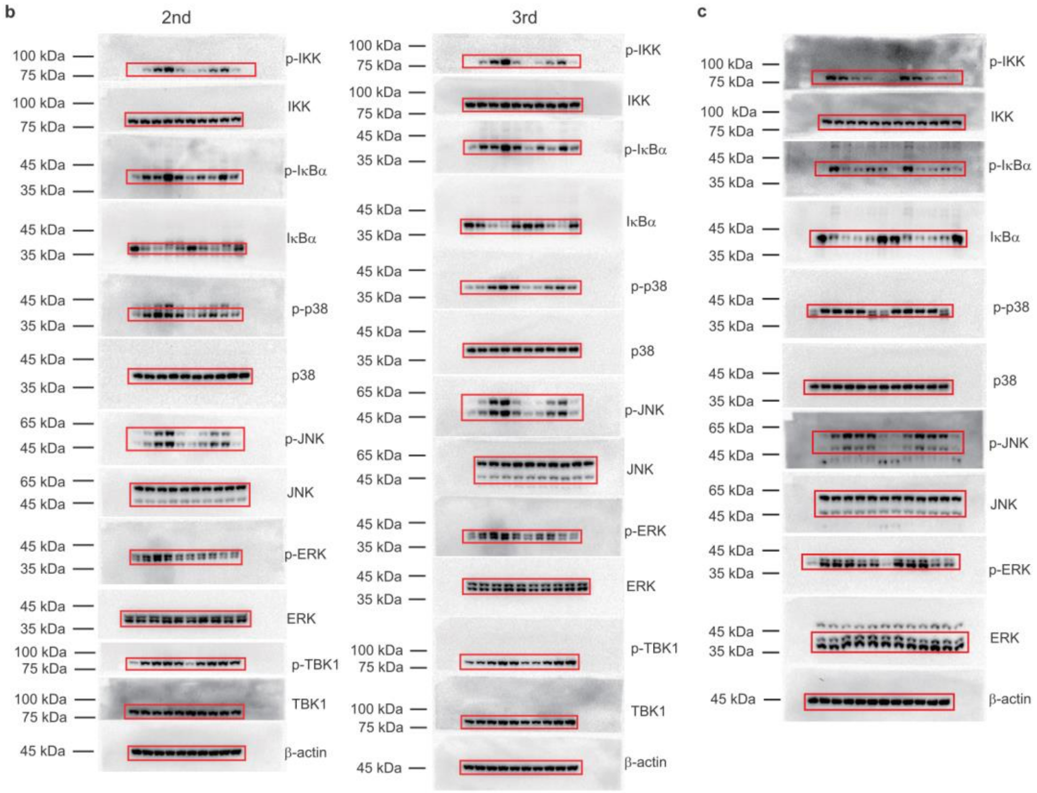

Supplementary Figure 14. Full-length uncropped western blots for supplementary figure 2. Cropped areas are marked by red box.

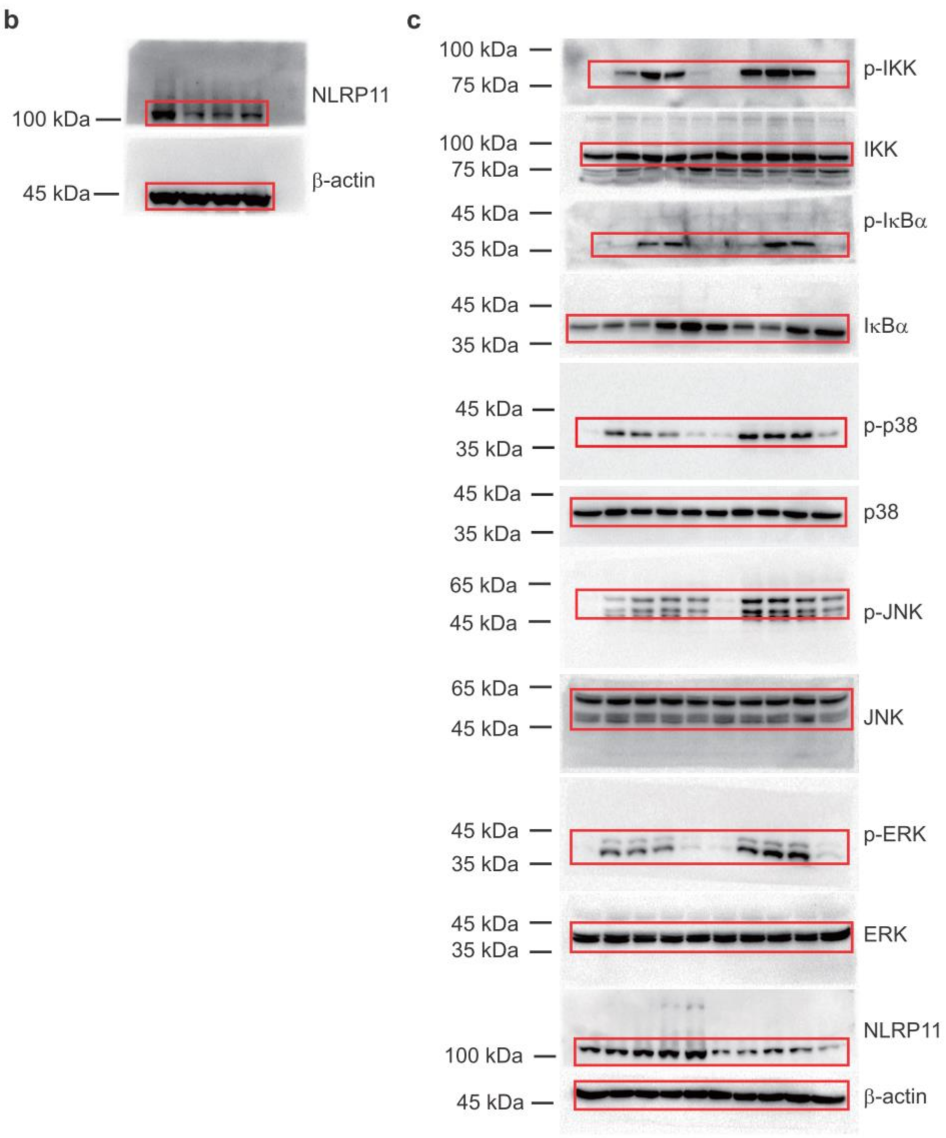

Supplementary Figure 15. Full-length uncropped western blots for supplementary figure 3. Cropped areas are marked by red box.

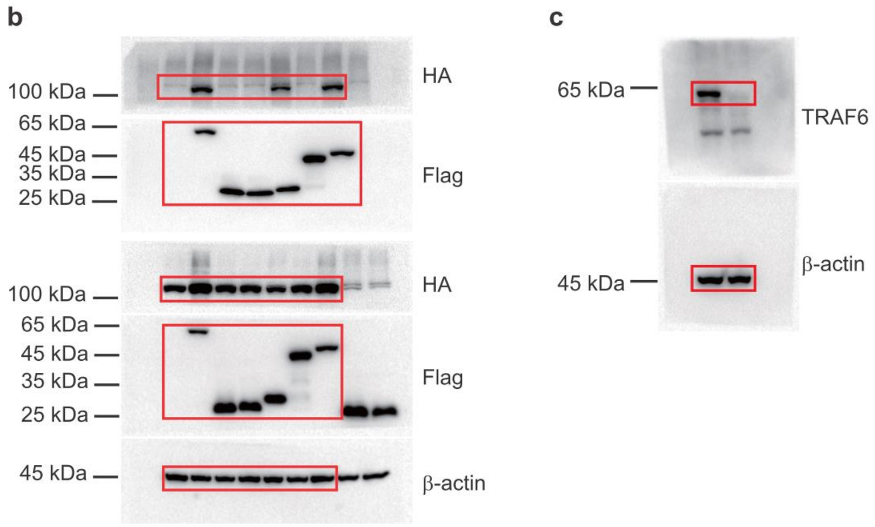

Supplementary Figure 16. Full-length uncropped western blots for supplementary figure 4. Cropped areas are marked by red box.

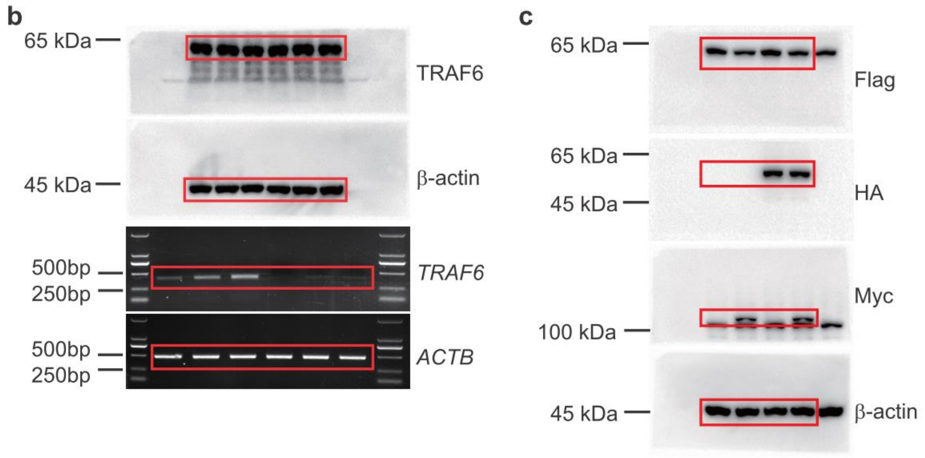

Supplementary Figure 17. Full-length uncropped western blots for supplementary figure 5. Cropped areas are marked by red box.

Supplementary Table 1. Primers used for real-time PCR

Gene name	Direction	Primer sequence (5'-3')
Human NLRP11	Forward	GGTCTTCTAAATGCAAACAG
	Reverse	CACTCAACTTAAGTGTCCTC
Human TNF	Forward	CCAGACCAAGGTCAACCTCC
	Reverse	CAGACTCGGCAAAGTCGAGA
Human IL-1β	Forward	ATGATGGCTTATTACAGTGGCAA
	Reverse	GTCGGAGATTCGTAGCTGGA
Human IL-6	Forward	AGAGGCACTGGCAGAAAACAAC
	Reverse	AGGCAAGTCTCCTCATTGAATCC
Human TRAF6	Forward	CAGGCTGTTCATAGTTTGAGC
	Reverse	CTGCTGTGCTTCGATTTCAG
Human β-actin	Forward	ACCATGTACCCTGGCATTGC
	Reverse	CGGACTCGTCATACTCCTGC
Human RNF19A	Forward	TGTGGCAACTGGGAACACTG
	Reverse	TCCACTTGCACTCAAACTGC
Human DTX4	Forward	GGAGTGGTCAAGCTACCCC
	Reverse	CTTCAGTGGGCCTCGAATGG
Human TRIM38	Forward	TGACGAACCCAGTAAGCATCAA
	Reverse	CTGCTCCGTACATCTGTCTTCA
Human RNF7	Forward	AATCCAGTGCCCTACAAAGG
	Reverse	AGCACTTCTAAGCTGTGTGC
Rhesus macaque NLRP11	Forward	GGTCTTCTAAATGAAAACAG
	Reverse	CACTCAACTTAAATGTCCTC
Rhesus macaque TNF	Forward	CCAGACCAAGGTCAACCTCC
	Reverse	CAGACTCGGCAAAGTCGAGA
Rhesus macaque IL-6	Forward	AGAGGCACTGGCAGAAAACAAC
	Reverse	AGGCAAGTGTCCTCATTGAATCC
	Forward	CCCACGAGCACTACAACGAG

Rhesus macaque IL-1β	Reverse	ACAAGCGTCGTTATTGCGTG
Rhesus macaque β-actin	Forward	ACCATGTACCCTGGCATTGC
	Reverse	GCTGATCCACATCTGCTGGA

Supplementary Table 2. siRNA sequence information.

siRNA	Direction	siRNA sequence
Human NLRP11 siRNA#1	sense	GCGAUAUCUGUCAAUAUAUTT
	anti-sense	AUAUAUUGACAGAUAUCGCTT
Human NLRP11 siRNA#2	sense	ACAUAAGAUUCGAGUUAAATT
	anti-sense	UUUAACUCGAAUCUUAUGUTT
Human NLRP11 siRNA#3	sense	CGACCUUGCAGCUGUCGAATT
	anti-sense	UUCGACAGCUGCAAGGUGGTT
Rhesus macaque NLRP11 siRNA#1	sense	GCACUCACAAAGACCGUUATT
	anti-sense	UAACGGUCUUUGUGAGUGCTT
Rhesus macaque NLRP11 siRNA#2	sense	CCAGCUGUUAUUCAGCCAATT
	anti-sense	UUGGCUGAAUAACAGCUGGTT
Rhesus macaque NLRP11 siRNA#3	sense	GCACGCAAGAUUCUGGAUUTT
	anti-sense	AAUCCAGAAUCUUGCGUGCTT
Human RNF19A siRNA#1	sense	CGGUGUUCCUAUUAUGUUATT
	anti-sense	UAACAUAAUAGGAACACCGTT
Human RNF19A siRNA#2	sense	GAUUCACAAUCGCUAUGAATT
	anti-sense	UUCAUAGCGAUUGUGAAUCTT
Human RNF19A siRNA#3	sense	CCUCAAUGAUAGCAGUUAATT
	anti-sense	UUAACUGCUAUCAUUGAGGTT
Human TRIM38 siRNA#1	sense	GGCCCUAUUUCCAGGUUUATT
	anti-sense	UAAACCUGGAAAUAGGGCCTT
Human TRIM38 siRNA#2	sense	CAGCAAUGCGAAUAACUAATT
	anti-sense	UUAGUUAUUCGCAUUGCUGTT
Human TRIM38 siRNA#3	sense	AGCUUUACUUCGAUGUGAATT
	anti-sense	UUCACAUCGAAGUAAAGCUTT

Supplementary Table 3. shRNA sequence information.

shRNA	Target sequence
TRIM26	TTCTCTGTGATCTCAACAG
LRSAM1	TAATCTGGCTCCTGATCTG
NFX1	ATCACTGAATTTTGACCCT
RNF12	AGTCTAAACTGGTAATTTC
SH3RF1	AATCTGCACAAAGTTGGTG
XIAP	TAAAGAAACCCTGCTCGTG
MNAT1	TTGGTGGTCTTACACCGAG
MYCBP2	AATGTGATGAAAGACGTTG
DTX4	TAGATGAGGGAGATGAGGG
ARIH1	TATGCAGGGCCATTCTCAG
MDM4	TATGTACTGACCTAAATAG
TRIM46	AAGTACTTCCTGGGCATAG
BAZ1B	TTCTGTATGGCACTCACTG
RNF19A	ATCACTAACTGTTCCCAAG
TRIM11	TATTATAGGACATCTACTC
TRIM58	TTCCTCATAAGTTCCAGAG
RNF157	TCTCGGTCTAAATCAAAGC
TRIM32	TCAAACATGGCACAAAGGG
MKRN3	AATACCTGCAGATGATCTG
TRIM6	TAAATGTAGAGAGGAAGGC
SYVN1	AATCATCTGAAACTGTCTC
RNF160	TTCATCAACTGTGGAATGC
CHD4	TATCCCATAGCGATAGAAG
ANKIB1	AAGAACACTTGAGAATGCG
RNF168	TTACTGAGCAGACGAACTG
BFAR	ATTGGGTTAAAGTACAGGG
PCGF3	AAATCTATCAGCTTAGCAC
PJA2	TACTTCACAGTTAAATGAG
WDSUB1	TTCAAATCATCAGCCAGAC
SFRS2IP	TATGGGAAGATATTCATAG
WHSC1L1	TAGTTTAATTTCAGGTGAG
TRIM2	TTCTCTGTGATCTCAACAG
TRIM33	TTGTGAAGTTCATAACATG
VPS8	TATCCATATGTACAATGAG
BAZ1B	TTCTGTATGGCACTCACTG
RAD18	TACCAGTTCATCTAATATG
ECHDC1	TTATACATATTAGTCACTG
RNF24	AATATAACCTGTTTGTAGG
PEX12	TAATAGATTTCCAAAGCTG
TRIM25	TAAATCCACTAACTGTTCC

BRCA1	TAAGGGACCCTTGCATAGC
TRIM54	TGATCAGCTCCTTGGCCTG
RFPL3	AATCGAATCAAATTAGAAT
EPM2A	TAATGCATGGCTTGGTGGC
TRIM5	TGCAGTGAGGCATGCTTGG
TTC3	AATCTTTATATTGCACACC
RNF2	AATATAACCTGTTTGTAGG
RNF213	AAGGAATGTATTTATCTAG

Other shRNA sequence can be found in the site of

http://dharmacon.gelifesciences.com/
